# Stem subsidence of polished and rough double-taper stems: In vitro mechanical effects on the cement-bone interface

**DOI:** 10.3109/17453670902967265

**Published:** 2009-06-01

**Authors:** Ayumi Kaneuji, Kengo Yamada, Kenichi Hirosaki, Masahiro Takano, Tadami Matsumoto

**Affiliations:** ^1^Department of Orthopaedic Surgery, Kanazawa Medical UniversityKahokugunJapan; ^2^Department of Machinery, Industrial Research Institute of IshikawaIshikawaJapan

## Abstract

**Background and purpose** Many clinical reports have indicated that polished hip stems show better clinical results than rough stems of the same geometry. It is still unknown, however, what the mechanical effects are of different surface finishes on the cement at the cement-bone interface. We compared mechanical effects in an in vitro cemented hip arthroplasty model.

**Methods** Two sizes of double-taper polished stems and matt-processed polished stems (rough stems) were fixed into composite femurs. A 1-Hz dynamic load was applied to the stems for 1 million cycles. An 8-h no-load period was set after every 16 h of load. Stem subsidence within the cement, and compressive force and horizontal cement creep at the cement-bone interface, were measured.

**Results** Compared to rough stems, stem subsidence, compressive force and cement creep for polished stems were a maximum of 4, 12, and 7-fold greater, respectively. There was a strong positive correlation between stem subsidence and compressive force for polished stems. In contrast, a strong negative correlation was found between stem subsidence and compressive force for rough stems. There was also a statistically significant relationship between compressive force on the cement and cement creep for the polished stems, but no significant relationship was found for rough stems.

**Interpretation** This is the first evidence that different surface finishes of stems can have different mechanical effects on the cement at the cement-bone interface. Stem subsidence in polished stems resulted in compressive force on the cement and cement creep. The mechanical effects that polished taper stems impart on cement at the cement-bone interface probably contribute to their good long-term fixation and excellent clinical outcome.

Many studies have shown that the long-term survival of a polished stem is better than that of a rough-surfaced stem of the same geometry in cemented total hip arthroplasty (THA) (Dall et al.1993, Howie et al. 1998, Meding et al. 2000, Collis and Mohler 2002). The good long-term results of polished taper stems are probably attributable to the preservation of the proximal femoral cortex and such stems are associated with a low incidence of radiolucent lines in the proximal femur (Fowler et al. 1988, Wroblewski et al. 2001, Yates et al. 2002). Furthermore, using finite element analysis, it has been shown that polished prostheses give limited stem subsidence and cement creep (Verdonschot and Huiskes 1996, 1997, 1998, Lu and McKellop 1997, Norman et al. 2001), which may be beneficial. This suggests that stem subsidence without cement fracture observed in clinical practice, a phenomenon specific to polished taper stems (Fowler et al. 1988, Howie et al. 1998, Yates et al. 2002, Williams et al. 2002, Ek and Choong 2005), may be attributable to cement creep (Weightman et al. 1987). In a taper stem scenario, Lee (1990) hypothesized that the forces applied to the cement, to the cement-stem interface, and to the bone-cement interface may differ depending on the surface finish of the stem. The forces applied due to a rough stem are mainly tensile and shear forces, while for a polished stem they are mainly compressive. Assuming that this hypothesis is correct, these differences in the force applied to the cement and bone may explain the difference in clinical results for polished and rough stems. Lee's theory has, however, not been studied using a mechanical model. It is difficult to perform a comparative study in cadavers since bone quality, bone shape, and femoral canal size vary—which may influence the results. Furthermore, the characteristics of cement can change as a result of changes in temperature and humidity (Lee et al. 1990, Arnold and Venditti 2001). For this study, we used an in vitro simulated cemented hip replacement model to quantify the difference in stem subsidence of polished and rough surface-finished taper stems into the cement and to determine whether the surface finish has an influence on the mechanical effects of any differences in the cement.

## Material and methods

### Femoral stem implantation

**Figure 1.**
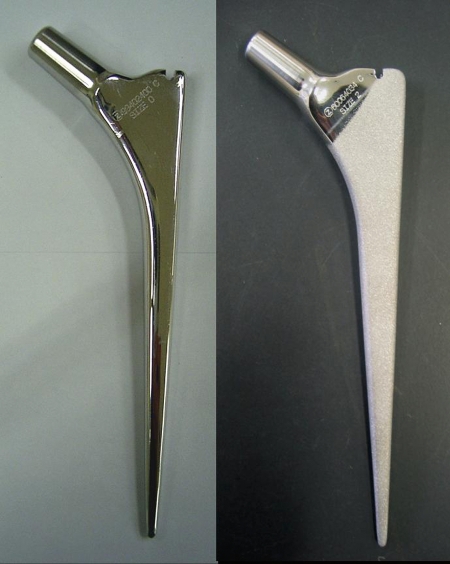
Collarless double-taper (CPT) stems. A polished stem is shown to the left and a matt-processed rough stem is shown to the right.

We used composite femurs (no. 3303; Pacific Research Laboratories, Vashon, WA), which are similar in shape, mechanical characteristics, and material density to those of human femurs (Cristofolini et al. 1996, Heiner and Brown 2001). 4 stems were tested. 2 polished stems (size 2 and 3) and 2 rough stems (size 2 and 3) were implanted into one size of composite femur. The polished stem was the collarless polished taper stem (CPT stem; Zimmer, Warsaw, IN) tapered in the coronal and sagittal planes and with a surface roughness of 0.1 μm or less. The rough stem, with a surface roughness of 5.291 (SD 1.100) μm, was processed from the CPT stem by blowing glass beads using an air-blast machine (DP-5; Fuji Seiki Co.Ltd., Shizuoka, Japan) (Figure 1). A centralizer, dedicated to the CPT stem, was attached to the stem tip. The proximal transverse diameter and offset of the size-3 stem were larger than that of the size-2 stem (by 2.5 mm and 1 mm, respectively).

### Mechanical testing

The composite femur neck was cut obliquely at 20 mm distal to the top of the greater trochanter and the distal part of the femur was cut at 230 mm from the top of the greater trochanter before attachment to the fixator. 8 pairs of 6-mm diameter holes were created in each of the medial, lateral, anterior, and posterior cortices of the femoral metaphysis and diaphysis. The uppermost hole was created at 10 mm distal to the femur neck cut. The distance between the upper and lower holes was 12 mm. A distance of 65 mm was provided between the proximal and distal parts. The upper holes were used for the placement of the rods containing the sensors to measure the compressive force, and the lower holes were used for placement of the rods containing the sensors to measure cement creep. After the holes were created, the composite femurs were immersed in blended vegetable oil for 24 h to simulate the humidity of an actual human femur.

### Fixator

**Figure 2.**
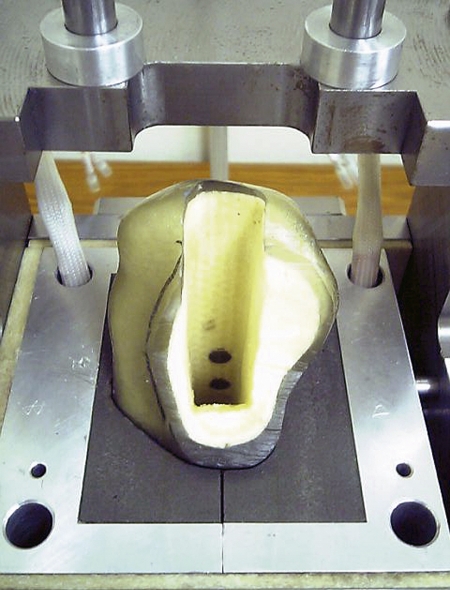
A composite femur fixed in the tester. Rods were set at the cement-bone interface.

A fixator constructed of machine-structural-use carbon steel and epoxy resin was made, to secure the composite femur during testing. The epoxy resin was formed to the contours of the composite femur. Holes were positioned on the fixator so that they were aligned with those in the composite femur during testing. Thus, the rod protecting the measuring equipment could penetrate both the fixator and the composite femur, enabling measurement at a constant site. The rod that passed through the inner tube was fixed to the face of the medullary canal of the composite femur (Figure 2) and vacuum-mixed cement (Osteo-bond; Zimmer) was injected with a cement gun.

A distal medullary plug was used before stem insertion. The stem was fixed with pressurized cement by 2 thumbs (the early cementing technique). After 24 h, the upper inner tube was replaced with the load cell, which was in direct contact with the cement-bone interface. The lower inner tube was removed. The temperature of the prepared femurs was maintained at 37oC using a heater and a temperature sensor attached to the epoxy resin.

### Load

A dynamic sine wave load of 3,000 N was applied over 1 million cycles at a frequency of 1 Hz to the metal head fixed to the stem at 15 degrees to the coronal plane (Bergmann et al. 1993) using a hydraulically controlled fatigue tester (Instron Japan Co. Ltd., Kanagawa, Japan). The load of 3,000 N is equivalent to the load applied to the hip joint when a person weighing 70 kg stands on one leg. 1 million cycles of load application corresponds to 1 year of walking (Crowninshield et al. 1980, Zahiri et al. 1998). Assuming sleep time, a no-load period of 8 hours was provided every 16-h period of load application. The total load period for a stem was 19 days.

### Mechanical assessment

**Figure 3.**
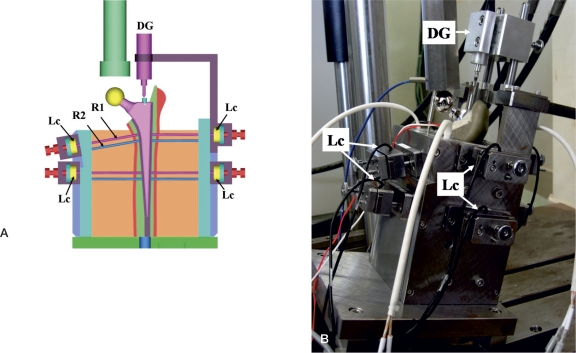
A. An illustration of the tester. The measuring rod (R1 and R2) penetrating the fixator and the femur was placed at the cement-bone interface. Compressive force was measured with the load cell (Lc) via the upper rod (R1) in each site. The amount of cement creep was measured via the lower rod (R2). Stem subsidence was measured with the digital dial gauge (DG) contacting the upper end of the stem. B. The actual tester. The inside was inclined 15 degrees from the base on the coronary plane so that the load was applied to the femur head attached to the top of the stem from 15 degrees inside.

Stem subsidence, and compressive force and cement creep at the cement-bone interface were measured (Figure 3). Stem subsidence was measured using a digital displacement gauge (DTH-A-5, 5 mm; Kyowa Electronic Instruments Co., Ltd., Tokyo, Japan) applied to the proximal lateral aspect of the stem. A positive value represented stem displacement in the distal direction. A load cell (TR20 I 500N/fs, TR20 I 200N/ fs; Kyowa) placed in the inner tube of the rod contacting the cement-bone interface was used to measure compressive force on the cement at this site. A positive value represented a horizontal force from the cement to the cortex.

Cement creep was measured from the displacement of the measuring rod outward with a dial gauge (TM-1205; Teclock Co., Ltd., Nagano, Japan). The measuring range of the dial gauge was 1 to 5,000 μm and the error of the device was 0.5 μm. The technical error was ± 0.577 μm when 2 people took 3 measurements each. The value of cement creep was defined as the difference in the amount measured by the measuring rod installed at the cement-bone interface before and after the experiment.

Stem subsidence and compressive force were measured over time and the data was automatically entered into a computer using measurement collection and analysis software (sensor interface PCD-300A, Kyowa). 10,000 data sets corresponding to approximately 8 min were stored as one file per 30 min and a total of 912 files were collected. The data obtained were corrected and converted to distance and pressure units.

The periods of load and no load in a day were classified as 3 categories: early, middle, and late. Stem subsidence in each period was defined as the mean of the values collected in the 2 consecutive files (20,000 data sets) after the start of each period. The compressive force in each period was defined as the mean of the 960 (60 values/min × 8 min × 2 times) maximum values of sine waves collected in the 2 consecutive files after the start of each period. 57 (3 × 19 days) averaged values were used for analysis of stem subsidence and compressive force, respectively.

The stress relaxation value (Niels 1980, Lee at al. 1990) was defined as the difference between the mean of the minimum forces of sine waves in the 2 files collected in the late load period and the mean force in the 2 files collected in the late no-load period immediately afterwards. The final compressive force and stress relaxation values were obtained as the mean (SD) of the values on the final experiment day.

### Statistics

The data were analyzed using SAS statistical analysis software (SAS Institute, Cary, NC). Inferential analysis was performed using the unpaired t-test and associations were examined using linear regression analysis and Pearson's correlation coefficient. A probability (alpha) of 5% was used to indicate statistical significance.

## Results

The stem and cement pistoned vertically during load with an amplitude of 1 Hz. This pistoning was independent of the composite femur. This phenomenon was independent of surface finish and was observed in all stems.

### Stem subsidence

The stem subsided only during the load period and it rose by a slightly smaller amount than the amount of subsidence during the no-load period (Table 1). All stems subsided progressively over time. The final stem subsidences for size-2 rough and polished stems were 0.274 mm and 1.179 mm, respectively. For size-3 rough and polished stems, the final subsidences were 0.334 mm and 0.521 mm, respectively. The subsidences were larger for the polished stem than for the rough stem: 4.3-fold with size 2 and 1.6-fold with size 3.

**Table 1.** Stem subsidence

	Load periods	No-load periods
Polished size 2	0.218 (0.082)	–0.148 (0.026)
Rough size 2	0.111 (0.023)	–0.094 (0.014)
Polished size 3	0.160 (0.022)	–0.141 (0.011)
Rough size 3	0.137 (0.018)	–0.124 (0.008)

The values represent the mean stem subsidence (in mm (SD)) in 1 day of 19 days. A positive value in average movement means downward direction of the stem, and a negative value means upward direction of the stem.

### Compressive force

The compressive force at the bone-cement interface, the force of the amplitude changed per 1 Hz during the load period. Such amplitude was not found during the no-load period. Differences in compressive forces among the measuring sites appeared as time elapsed, and relatively greater values were observed in the proximal medial and distal lateral portion. The compressive forces were relatively small in all other sites, including the anterior and posterior surfaces but excluding the proximal lateral site in the polished size-2 stem.

**Figure 4.**
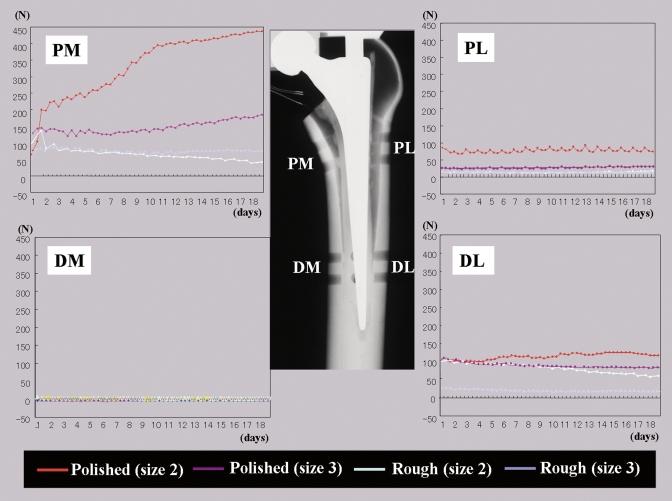
Compressive forces on the bone-cement interface. PM: proximal medial site; PL: proximal lateral site; DM: distal medial site; DL: distal lateral site.

In the proximal medial site, for which the compressive force was observed most clearly, the force increased in the polished stem as time went on. However, the force remained low in the rough stem and it decreased in the size-2 rough stem (Figure 4). The compressive forces applied to the bone-cement interface on the final day were higher in the polished stem than in the rough stem, 12-fold in size 2 and 3.3-fold in size 3. Smaller compressive forces or reduction in compressive forces were also observed for the rough stem in the distal lateral sites. The compressive forces applied to the distal lateral sites were higher in the polished stem than the rough stem, by 2.9-fold in size 2 and 3.1-fold in size 3 (Table 2).

**Table 2.** Final compressive force and cement creep at the cement-bone interface

		Proximal–medial	Distal–medial	Proximal–lateral	Distal–lateralt	Proximal–anterior	Distal–anterior	Proximal–posterior	Distal–posterior
Polished size 2	force	435 (1.7)	1.7 (0.1)	78 (6.5)	117 (0.3)	4.4 (0.3)	2.2 (0.1)	2.7 (0.2)	11 (0.7)
	creep	215	1.0	27	152	–4.0	–14	–0.3	35
Rough size 2	force	36 (2.2)	–1.0 (0.1)	17 (1.1)	58 (2.3)	3.5 (0.5)	1.3 (1.1)	2.0 (0.3)	4.4 (1.1)
	creep	29	–33	11	46	26	10	8.0	22
Polished size 3	force	179 (3.7)	–1.3 (0.3)	29 (0.4)	75 (1.1)	5.2 (0.2)	1.9 (0.3)	9.0 (1.4)	–6.6 (0.5)
	creep	90	–4.0	16	23	–1.0	–0.3	11	–19
Rough size 3	force	55 (3.0)	1.0 (0.1)	16 (0.1)	24 (1.2)	8.4 (0.6)	0.8 (0.1)	3.4 (0.4)	4.7 (1.6)
	creep	34	–19	20	10	–0.3	–1.0	–0.3	3.6

The final compressive force is the mean value (SD) of the 960 times of maximum values of 1 Hz sine wave of the late period in the final experiment day, and the cement creep was the mean value (SD) of three times measurements in each site. The values are the average N (SD) of the force and the average micron meter (SD) of the cement creep.

**Figure 5.**
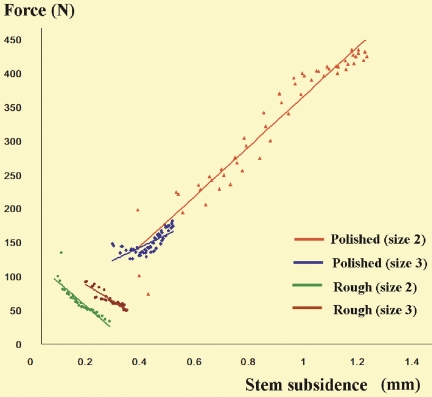
Stem subsidence and compressive force at the cement-bone interface. Stem subsidence in each period was defined as the mean of values in the two consecutive files (20,000 data sets) after the start of each period. The compressive force in each period was defined as the mean of the collected 960 maximum values of sine waves in the two consecutive files after the start of each period. 57 (3 periods x 19 days) averaged values were used for analysis of stem subsidence and the compressive force, respectively. Simple regression analysis, significances, and correlation coefficients (r): P2; y = 369.44x – 3.5987, R2 = 0.935, p < 0.001, r = 0.9667. P3; y = 191.92x + 66.66, R2 = 0.536, p < 0.001, r = 0.7322. R2; y = –347.08x + 128.55, R2 = 0.779, p < 0.001, r = 0.8837. R3; y = –244.51x + 138.91, R2 = 0.8633, p < 0.001, r = 0.9291. y: force; x: stem subsidence, R2 = r2.

A statistically significant regression was observed between the stem subsidence and compressive force at the proximal medial site with all stems. A significant positive correlation was found between stem subsidence and compressive force in the polished stems while a significant negative correlation was found in the rough stems. This suggests that subsidence of the polished stem may lead to an increase in compressive force at the bone-cement interface while subsidence of the rough stem may induce a decrease in compressive force (Figure 5).

### Cement creep

The amount of cement creep was appreciable at the sites where large compressive forces were observed: proximal medial and distal lateral sites. The cement creep was larger in the polished stem than in the rough stem, by 7.4-fold and 2.6-fold in size 2 and 3 stems at the proximal medial site and by 3.3-fold and 2.3-fold in size 2 and 3 stems at the distal lateral site (Table 2). The greatest degree of cement creep (of 215 μm) was observed at the proximal medial site of the size 2 polished stem.

In the simple regression analysis, a statistically significant relationship between the compressive force (x) and cement creep (y) of 4 proximal and 4 distal sites was found in the polished stems (size 2: y = 0.672x + 8.085, R^2^ = 0.822, p < 0.002; size 3: y = 0.520x – 0.852, R^2^ = 0.887, p < 0.001) while it was not significant in the rough stems (size 2: y = 0.487x + 4.832, R^2^ = 0.449, p = 0.07; size 3: y = 0.337x – 0.967, R^2^ = 0.407, p = 0.09). This suggests that the compressive force caused cement creep in the polished stems.

### Stress relaxation

**Figure 6.**
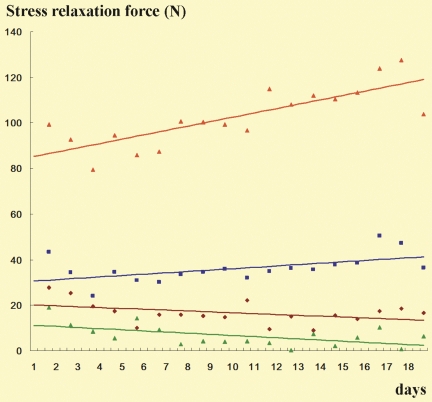
Stress relaxation at proximal medial site. Color codes, see Figure 5. The stress relaxation value in a day was defined as the difference between the mean of the 960 minimum forces of sine waves in the 2 files collected in the late load period and the mean of the 960 minimum forces in the 2 files collected in the late no-load period. A total 19 values were collected for data in each stem.

Stress relaxation occurring between the late load period and the late no-load period, namely, the amount of relaxation of the stress accumulated in the cement, was evaluated in the proximal medial site where the compressive force was largest. The amount of stress relaxation tended to increase over time in the polished stems whereas it did not increase in the rough stems (Figure 6). The stress relaxation value on the final day was 104 (SD 2.2) N and 37 (SD 2.2) N in the polished stems (sizes 2 and 3, respectively) whereas it was 6.4 (SD 1.2) N and 17 (SD 1.1) N in the rough stems. Thus, the final stress relaxation was larger in the polished stems than in the rough stems by 16-fold in size 2 and 2.2 -fold in size 3.

## Discussion

We found that the surface finish of the cemented stem creates a difference in the dynamic behavior. In the polished stems, there were correlations between stem subsidence and degree of compressive force and cement creep. We suggest that subsidence of the stem gradually presses the cement toward the periphery of the medullary canal, which leads to cement creep. Thus, compressive force tends to be applied to the cement-bone interface, which support a theory suggested in the past (Fowler et al. 1988, Lee 1990, Yates et al. 2002). In contrast to the polished stem, we found a negative correlation between stem subsidence and compressive force in the rough stem. This suggests that the shear force on the bone-cement interface pulls the cement toward the inside of the medullary canal, and we speculate that stem subsidence of the rough stem tends to cause the reduction in compressive force on the cement-bone interface.

We have found no earlier reports in which cement creep was measured in a THA model. In our study, we used vacuum-mixed cement that is used in clinical practice, which may enhance cement durability (Alkire et al. 1987, Wixson et al. 1987). Although Norman et al. (1995) reported that vacuum mixing reduces creep strain by 48%, we found a maximum of 215 μm of cement creep using vacuum-mixed cement, which suggests that similar cement creep occurs in vivo. Kaneuji et al. (2006) reported that improvement of radiolucency in the CPT stem is prominent in Gruen zone 7. We also found larger compressive force and creep in zone 7.

Stress relaxation is defined as a change in stress level over time at constant strain (Niels 1980, Lee 1990). In other words, stress relaxation is considered to be the decrease in strain energy accumulated in cement that has crept by stem subsidence. Greater relaxation of strain energy can result in greater ability in self-protection against fatigue breakage (Niels 1980, Lee 1990). Clinically, the opportunity for strain energy relaxation such as during sleep is important, as the stress relaxation tends to occur during the no-load period (Lu and McKellop 1997). The stress relaxation was reproduced in our THA model, and the pattern in the polished stems was different from that in the rough stems. We found that stress relaxation remains high over time in the polished stem. This may be one of the reasons for there being less failure with the polished stem in clinical practice.

Clinical radiostereometric analyses and reports on retrieved femoral implants (Alfaro-Adrian et al. 1999, Howell et al. 2004), and also the report on the mechanical experiment that evaluated stem subsidence and micromotion in THA models (Ebramzadeh et al. 2004), suggest that subsidence and micro-motion can also be found in the unloosened rough stem, though the amount is smaller compared to the polished stem. The polished stem is less likely to damage the cement interface when it moves in synchrony with amplitude (Howell et al. 2004). The mirror-like surface of the polished stem enables the cement to fit perfectly, allowing no micro-gap and no space for debris passage (Crawford et al. 1999). On the other hand, repeated micromotions in the matt finished rough stem could cause micro-fracture of cement and cement debris, and produce a passage for wear particles down to the femoral canal (Crawford et al. 1999, Howell et al. 2004). We were able to show the occurrence of micromotion. Although the final amount of stem subsidence was larger in the polished stem than in the rough stem, daily subsidence and rise in the rough stem were more than 0.1 mm and almost the same as those for the polished stem. One possible cause of difference in the amount of final stem subsidence was that the stem tended to return to the original position during the period with no load. Each stem has a hollow centralizer at the bottom, to allow for stem slip without cement breakage. Polished stems slipped in the cement and centralizer during loading, and the stems tend to maintain their position through cement creep and stress relaxation. However, rough-surface stems do not slip easily in the cement and centralizer because of micro-bonding of the rough surface with the cement, and the stems tend to return to their original position during the periods of no load. As a result of this difference, we consider the centralizer to be effective for polished stems, but less effective for rough stems.

The forces at the proximal medial and distal lateral sites were stronger than at other sites, probably because the load was applied to the stem in our model only in one direction in the coronal plane. In other words, the load was transmitted to the calcar region and the moment was reflected as the counter reactive force on the distal lateral site.

The small stem had a thicker cement mantle than the large one, because same size of composite femur was used for all stems. More subsidence and compressive force were seen in the small-size polished stem than in the large one. This may indicate that a small stem is likely to subside into cement more than a large one. However, we cannot conclude this because we only studied a small number of stems. We believe that the differences in results according to stem size in each surface-finish group had some range of variation. However, our study indicates differences between polished and rough surface stems because the results in each surface finish group showed a similar pattern (but a different pattern in the other one). The correlation between stem subsidence and compressive force is the most important and obvious difference. Each final value of stem subsidence and compressive force is the mean value of a large amount of data with a small range of standard deviation. Thus, we believe that the same pattern in the same surface finish group and the opposite pattern in the other surface finish group indicated a real difference, even with the small number of stems.

The most important finding of our study was that cement creep, stress relaxation, and reverse subsidence occur during the periods of no load. These have not been measured in previous studies. However, it was impossible to measure the shear force, i.e. vertical force, with the load cell. If the shear force is measured, the mechanism of loosening can be explained more easily.

Future studies should include a long-term comparison of more stems, loading in different directions, and trying to measure shear stress at the interface. However, the theoretically predicted behavior of the cemented collarless double-taper stem was verified in our study and the results may lead to a better understanding of loosening mechanisms in cemented stems.
